# A Reinforcement Learning-Based Strategy of Path Following for Snake Robots with an Onboard Camera

**DOI:** 10.3390/s22249867

**Published:** 2022-12-15

**Authors:** Lixing Liu, Xian Guo, Yongchun Fang

**Affiliations:** Institute of Robotics and Automatic Information System, College of Artificial Intelligence, Nankai University, Tianjin 300071, China

**Keywords:** snake robots, visual localization, path following, reinforcement learning control

## Abstract

For path following of snake robots, many model-based controllers have demonstrated strong tracking abilities. However, a satisfactory performance often relies on precise modelling and simplified assumptions. In addition, visual perception is also essential for autonomous closed-loop control, which renders the path following of snake robots even more challenging. Hence, a novel reinforcement learning-based hierarchical control framework is designed to enable a snake robot with an onboard camera to realize autonomous self-localization and path following. Specifically, firstly, a path following policy is trained in a hierarchical manner, in which the RL algorithm and gait knowledge are well combined. On this basis, the training efficiency is sufficiently optimized, and the path following performance of the control policy is greatly improved, which can then be implemented on a practical snake robot without any additional training. Subsequently, in order to promote visual self-localization during path following, a visual localization stabilization item is added to the reward function that trains the path following strategy, which endows a snake robot with smooth steering ability during locomotion, thereby guaranteeing the accuracy of visual localization and facilitating practical applications. Comparative simulations and experimental results are illustrated to exhibit the superior performance of the proposed hierarchical path following the control method in terms of convergence speed and tracking accuracy.

## 1. Introduction

Over the past decades, many researchers have been devoted to the control of snake robots due to their complex multi-joint structure and high motion flexibility [1,2,3]. As the application tasks of snake robots become more complex, the requirement for accomplishing a safe and accurate path following tasks with independent perception continues to increase. Specifically, path following, as one of the most fundamental and indispensable motion skills, requires the robot to move along a specific curve. Visual self-localization provides the real-time position of the robot via visual perception, which plays an important role in assisting a robot in completing autonomous motions. However, the highly redundant degrees of freedom and unique serpentine motion gait introduce many challenges to the path following of snake robots with visual self-localization.

Snake robots typically move forward by mimicking the motion gait of biological snakes. One of the most efficient and widely used motion gaits is the lateral undulatory gait; that is, it periodically propagates a wave along the body, presenting an S-shaped movement trajectory, which is named a serpenoid by Hirose [1]. For snake robots, the lateral undulatory gait can be mathematically approximated by a gait equation that imposes a sinusoidal signal for each joint. The path following control of snake robots has been investigated for years based on the gait equation. There is substantial research that focuses on designing a control law for the gait equation to adjust the motion direction and thus control the robot in moving towards the desired path with the desired turning angle, which is calculated by the line-of-sight (LOS) guidance law [4,5,6]. Furthermore, for more complicated application scenarios, path following controllers with an adaptive LOS guidance law or gait equation are adopted for faster convergence speed, and higher stability [7,8,9]. In addition, for holonomic snake robots, virtual constraints are employed to regulate the orientation, and forward speed of the snake robot via a state-dependent undulatory gait equation, which replaces time-dependent signals in the lateral undulatory gait equation with state-dependent constraints [10,11,12,13]. However, the methods mentioned above heavily rely on precise modelling and laborious parameter tuning, which cannot guarantee optimal path following performance.

Reinforcement learning (RL), as a popular machine learning algorithm that constantly interacts with the environment to maximize expected returns, has made much progress in robotic control [14,15,16]. Unlike traditional control methods, RL algorithms endow robots with various motion skills without acquiring the exact robot model and exhibit excellent robustness and flexibility regarding environment variations. In addition, in RL, the control objectives and constraints can be conveniently added as terms to the reward function to guide robots to complete the specified task, e.g., manipulator manipulation, tracking a target velocity, mimicking human motor skills, etc. Model-free (MF) RL has shown its unique advantages in mastering specific skills or accomplishing specific tasks. However, end-to-end MFRL training often suffers from sample inefficiency and is prone to generating weird and unnatural actions, which seriously reduce training speed, and the learned policy may even damage the practical robot. Consequently, it is essential to integrate RL algorithms with gait knowledge to improve training efficiency and generate natural actions that make deployment on a practical robot easier. Recently, RL algorithms assisted by gait knowledge have made great progress in the fields of quadruped robots, bipedal robots, etc. [17,18,19,20,21]. For snake robots, the gait equation can reflect the shape of the motion trajectory; thus, it is an ideal source of gait knowledge. However, incorporating the gait equation with the RL algorithm for path following tasks is still a difficult problem. In our prior work [22], a two-stage control framework that combines PI^2^ with the gait equation is proposed for the snake robot to perform goal-driven tasks, but the gait equation is only adjusted at each gait cycle. As a result, the robot cannot promptly correct its gait according to the position error, so the control accuracy of this method is too low for performing path following tasks.

In addition to path following control, satisfactory visual self-localization of snake robots is also a challenging topic. Due to the slender body structure and serpentine motion gait, a snake robot obtains forward momentum using whole-body motion; thus, the camera installed on the robot also shakes accordingly, which brings difficulties to stable visual perception and localization. To solve this problem, the robot is usually required to remain stationary or to move slowly during the imaging process, which optimizes localization accuracy but sacrifices locomotion efficiency [23,24]. Ref. [25] proposes a pan-tilt compensation strategy to realize visual self-localization without reducing the robot’s locomotion efficiency, in which the position of the robot is updated by an external visual marker. The camera is mounted on a pan-tilt that actively rotates to compensate for head motions, which ensures that the visual marker is always within the camera’s field of view. Consequently, this method requires relatively smooth head swings at adjacent moments to guarantee the successful compensation of the pan-tilt. However, during the training of the path following policy using RL algorithms, a snake robot is prone to learning a policy that gains a higher reward for approaching the desired path faster, leading to an aggressive policy; i.e., it vigorously wiggles the entire body from side to side to gain stronger forward momentum, based on which, the pan-tilt cannot promptly compensate for the motion of the head; thus, the camera installed on the pan-tilt will lose the target marker.

To solve the above-mentioned problems, a novel hierarchical path following the control method is proposed for snake robots, which presents high training efficiency and promising tracking performance. Specifically, the hierarchical path following the control method is developed by combining the RL algorithm and the gait equation. On the one hand, the hierarchical control method generates motion gaits by modifying the gait equation, which provides gait knowledge for the RL algorithm and thus accelerates the training process. In addition, it ensures that the resulting motor gait belongs to the lateral undulatory gait so that the learned policy can be directly transferred to the practical snake robot without any retraining. On the other hand, the proposed hierarchical method adjusts the gait equation in real-time via the RL algorithm, which expands the feasible gait set of the gait equation, thus enabling a snake robot to change the motion gait based on the feedback state promptly. In addition, in order to enable a snake robot to achieve visual self-localization using the pan-tilt compensation strategy, a visual localization stabilization item is added to the reward function of RL policy training, which effectively limits the swing amplitude of the head at adjacent moments. The contributions of this paper are summarized as follows:

- A novel hierarchical control method that combines the RL algorithm and the gait equation is developed for the path following of snake robots, which guarantees efficient training and is satisfactory following the accuracy.
- A visual localization stabilization term is introduced into the reward function to avoid excessive head swings, which ensures successful pan-tilt compensation, thereby optimizing the accuracy of visual localization.
- To verify the effectiveness of the algorithm, real-world experiments are implemented on a practical snake robot, and the experimental results demonstrate the promising path following the performance of the proposed method.

## 2. Materials and Methods

### 2.1. Problem Statement

The path-following task of snake robots can be formulated as a Markov decision process (MDP) defined by the tuple (S,A,R,P,γ), where S denotes the state space, A represents the action space, R is the reward function, P indicates the state transition probability, and γ stands for the discount factor. At each timestep, the snake robot samples and then executes the action $a_{t}∼\pi \left(a_{t}|s_{t}\right)$ based on current state $s_{t}$ observed from the environment, and then the robot transfers to a new state $s_{t+1}∼p\left(s_{t+1}|s_{t},a_{t}\right)$ and receives a reward $r_{t}=r\left(s_{t},a_{t},s_{t+1}\right)$, where the subscript ${⋆}_{t}$ depicts the current timestep *t*. The objective of this MDP is to train a policy $\pi_{\psi }^{*}$ with parameter ψ that maximizes the expected cumulative discounted return J, as indicated in Equations (1) and (2), so as to equip a snake robot with the excellent path following skill:

(1) $$\pi_{\psi }^{*}=\arg\max_{a\in A}J$$

(2) $$J=E_{\tau ∼p(\tau |\pi )}\;\left[\sum_{t=0}^{T-1}\gamma^{t}r_{t}\right]$$

where *T* denotes the planning horizon of each episode, and τ denotes a trajectory $\left\{s_{0},a_{0},r_{0},s_{1},\ldots ,s_{T-1},a_{T-1},r_{T-1},s_{T}\right\}$.

### 2.2. Hierarchical Path Following Control

In this paper, the objective is to design a controller that enables a snake robot to follow the given path with visual self-localization. To this end, we proposed a hierarchical RL path following method to guarantee satisfactory following the ability for various desired paths in terms of efficient training, strong robustness, and excellent following accuracy. The proposed hierarchical algorithm effectively incorporates the RL algorithm with the gait equation and consists of two layers, namely the RL policy training layer and the gait execution layer. Specifically, compared with the motion gait produced by the traditional gait equation, the proposed method tends to generate a forward gait with slighter head swings, which improves the accuracy of visual localization and further guarantees satisfaction following accuracy. In addition, compared with the end-to-end RL algorithm, the designed hierarchical RL method not only greatly accelerates the training speed but also learns a natural and robust policy that can be directly implemented on a practical snake robot.

**Figure 1:**
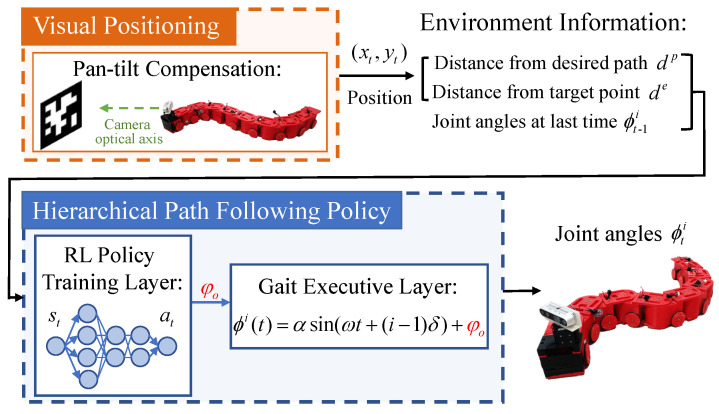
The overall control architecture of the proposed path following method, which consists of two stages: visual self-localization and hierarchical path following control.

The overall architecture of the proposed control method is presented in Figure 1, which consists of two stages, namely the visual localization and hierarchical RL path following policy training. Specifically, for a *n*—link snake robot, at each timestep *t*, firstly, the pan-tilt compensate strategy proposed in [25] is adapted to provide the real-time position of a snake robot, which can be used to obtain the current system state; subsequently, the RL policy training layer outputs an action to modify the gait parameter of the gait equation based on the state, with the aim of changing the motion direction of the robot to make it close to the desired path. Finally, the gait execution layer sends the corresponding joint angles to the snake robot for executing the path following task.

#### 2.2.1. Visual Localization

Due to the head swings caused by the lateral undulatory gait, the camera mounted on the robot head always loses the visual marker, so the position of the robot cannot be updated in real-time. Therefore, Ref. [25] proposes a pan-tilt compensation strategy to always keep the camera plane parallel to the visual marker plane via active compensation, where the compensation angle of the pan-tilt $\theta_{t}^{PT}$ is represented as follows:

(3) $$\theta_{t}^{PT}=-\theta_{t}^{head}$$

where $\theta_{t}^{head}$ is the orientation angle of the head of a snake robot at timestep *t*, and the compensation angle $\theta_{t}^{PT}$ is only related to $\theta_{t}^{head}$ with the same value and the opposite direction; that is, if the head turns to the left, the pan-tilt automatically rotates the same angle to the right to keep the camera facing the visual target. $\theta_{t}^{head}$ can be indicated in the following manner:

(4) $$\theta_{t}^{head}=\frac{\pi }{2}-\theta_{t}^{ac}-\theta_{t-1}^{PT}$$

where $\theta_{t}^{ac}$ denotes the current deviation angle between the visual marker plane and the camera plane after the last pan-tilt compensation. After the pan-tilt compensation, the position of the head of a snake robot can be calculated through visual localization and coordinate transformation as follows:

(5) $$\begin{array}{cc} X_{cam}^{w} & =R_{tgt}^{w}X_{cam}^{tgt}+p_{tgt}^{w}\\ X_{head}^{w} & =R_{cam}^{w}X_{head}^{cam}+X_{cam}^{w} \end{array}$$

where $X_{cam}^{w}$ is the coordinate of the camera mounted on the head, which can be calculated by rotation matrix $R_{tgt}^{w}$ and translation matrix $p_{tgt}^{w}$ from the visual target coordinate system to the world coordinate system and the position of camera $X_{cam}^{tgt}$ in the visual target coordinate system. Furthermore, $X_{head}^{w}$ is the position of the head of the snake robot, $R_{cam}^{w}$ denotes the rotation matrix between the camera coordinate system and the world coordinate system, and $X_{head}^{cam}$ expresses the coordinate of the head in the camera coordinate system.

Based on the above introduction, it can be derived that the angle of the robot head plays an important role in determining the accuracy of the visual localization. Excessive head swings lead to loss of visual markers, which further results in the failure of path-tilt compensation. Consequently, a visual localization stabilization term is proposed to reduce head swings during motion and embedded in the training process of the path following strategy, which will be described in detail below.

#### 2.2.2. Rl Policy Training Layer

As the first stage of the hierarchical control framework, the training objective of the RL policy training layer is to find a policy that outputs the optimal action according to the current state of the whole system, which will be used to modify the gait equation [1] in the later control stage to ensure good path following performance. To this end, the high-level control law of the hierarchical control framework is defined as follows:

(6) $$u_{high}=a_{t}∼\pi_{\psi }\left(a_{t}|s_{t}\right)$$

In order to achieve satisfactory path following performance, a snake robot is expected to approach the desired path as close as possible; in addition, to prevent the robot from stopping as soon as it approaches the path, random target points are selected on the desired path along the forward direction of the robot to guide its forward motion while continuing the path following.

The state of the path following task is embedded in vector $s_{t}\in R^{n+1}$, which consists of the distance between the real-time position of the snake robot and the desired path $d^{p}$, the distance between the robot and the endpoint $d^{e}$, and the joint angles command $\phi_{t-1}^{i},i=1,2,...,n-1$ at the last timestep.

According to state $s_{t}$, action $a_{t}\in R^{1}$ drives the snake robot to follow the desired path by adjusting the parameter of the gait equation, which generates the lateral undulatory gait as the Equation (7), with α, ω, δ, and $\phi_{o}$ denoting the gait amplitude, angular frequency, phase difference, and offseting of the lateral undulatory gait, respectively.

(7) $$\phi^{i}\left(t\right)=\alpha \sin\left(\omega t+\left(i-1\right)\delta \right)+\phi_{o}$$

where $\phi^{i}\left(t\right),i=1,2,...,n-1$ is the *i*-th joint angle of the snake robot at time *t*. Different groups of four gait parameters generate different forms of the motion trajectory of the lateral undulatory gait. To drive the robot to follow the desired path, the joint offset $\phi_{o}$, which can modify the motion direction in real-time, is selected as the action and then generated by the policy network.

(8) $$a_{t}=\phi_{o}$$

To improve the accuracy and efficiency of the path following, the reward function is designed as follows:

(9) $$r_{t}=r_{p}+r_{e}-p_{h}$$

where $r_{p}$ encourages the snake robot to approach the desired path with a defined tolerance, the second term $r_{e}$ rewards the robot for moving forward towards the endpoint as soon as possible, and the last term $p_{h}$ is the visual localization stabilization term, which penalizes the robot for excessive head swings in adjacent moments. Specifically, the three terms are constructed as follows:

(10) $$r_{p}=\left\{\begin{array}{cc} c_{p} & \;\mathrm{if}\;\left|d_{t+1}^{p}\right|<d_{1}\\ c_{p}\exp\left(d_{1}-\left|d_{t+1}^{p}\right|\right) & \;\mathrm{if}\;d_{1}\leq \left|d_{t+1}^{p}\right|\leq d_{2}\\ 0.0 & \;\mathrm{if}\;\left|d_{t+1}^{p}\right|>d_{2} \end{array}\right.$$

(11) $$r_{e}=c_{e}\left(d_{t}^{e}-d_{t+1}^{e}\right)$$

where $c_{p}$ and $c_{e}$ are the weighting constants, and $d_{1}$ and $d_{2}$ are the distance thresholds at which the reward approaching the goal path can be obtained.

In order to improve the accuracy of the visual localization mentioned in Section 2.2.1, the visual localization stabilization term $p_{h}$ is depicted as follows, with $c_{h}$ being a negative constant and $\phi_{*}$ being the angle threshold:

(12) $$p_{h}=\left\{\begin{array}{cc} c_{h} & \;\mathrm{if}\;\left|\phi_{t+1}^{1}-\phi_{t}^{1}\right|\geq \phi_{*}\\ 0.0 & \;\mathrm{if}\;\left|\phi_{t+1}^{1}-\phi_{t}^{1}\right|<\phi_{*} \end{array}\right.$$

where $\phi_{t}^{1}$ and $\phi_{t+1}^{1}$ are the head angles of the snake robot at timestep *t* and t+1.

The Proximal Policy Optimization (PPO) algorithm is adopted to train policy $\pi_{\psi }$, which is represented by a fully connected network with 3 Tanh hidden layers of [64,32,32] units.

#### 2.2.3. Gait Executive Layer

The gait executive layer is the second stage of the hierarchical control framework, which is controlled by the high-level action $a_{t}$ and presents a modified motion gait via the gait equation shown in Equation (7). The low-level control law is illustrated as follows:

(13) $$u_{low}=\phi^{i}\left(t\right)$$

The generated control command $u_{low}$ of the joint angels is directly sent to a snake robot and helps it to change the motion direction and then converge to the desired path.

## 3. Results

In this section, the hierarchical path following policy is firstly trained and then tested in the simulation, and the training efficiency and the effectiveness of the proposed algorithm are verified. Subsequently, the trained policy is directly transferred to real-world experiments, and several experiments are implemented on a practical snake robot to validate the actual following performance for different desired paths.

### 3.1. Simulations

**Figure 2:**
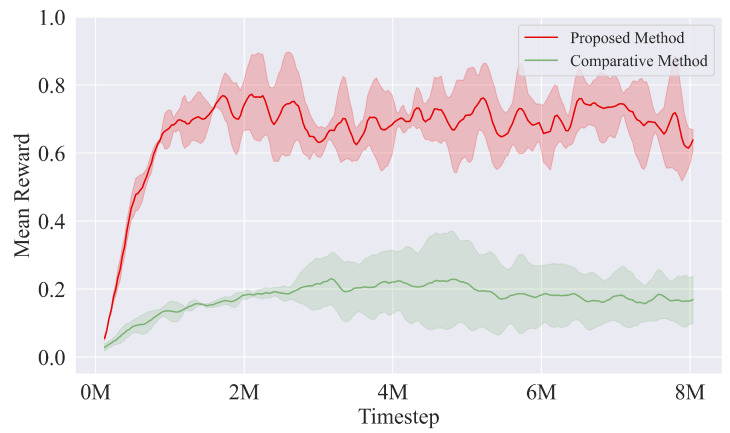
Mean reward of an episode for the proposed hierarchical method and the end-to-end comparative method.

The simulation environment is developed based on the Mujoco [26] simulator, and the model of the snake robot is composed of nine connection modules with a pair of passive wheels and eight yaw joints. During training, the start point of the snake robot is (0m,0m). The desired paths are straight lines $y=y^{*}\in \left[-1.5\;m,1.5\;m\right]$, sinusoidal curves y=Asinωx+ϕ, A∈[0.2m,1.0m], $\omega \in [\frac{\pi }{2}\mathrm{rad},\pi \mathrm{rad}]$, ϕ∈[−1.5m,1.5m], and circles $x^{2}+y^{2}=R^{2},R\in \left[1.5\;m,3.0\;m\right]$, respectively. The target point is a random point on the desired path with an *x*—coordinate $x^{*}\in \left[4.0\;m,5.0\;m\right]$. The end-to-end PPO algorithm, which takes the same state as the input and directly outputs joint angles, is selected as the comparative method to demonstrate the training efficiency and tracking performance of the proposed algorithm. Firstly, the comparison of the mean reward of an episode between the two methods is depicted in Figure 2.

The training results demonstrate that the proposed hierarchical control method achieves a superior training performance with a higher episode reward and faster convergence speed compared with the comparative method. Specifically, the proposed method converges to higher episode reward within about 1M timesteps, while the comparative method slowly converges to a reward value that is less than one-third of that of the proposed method at about 2M timesteps, which clearly indicates the strong path following ability and reliable training efficiency of the proposed method.

**Figure 3:**
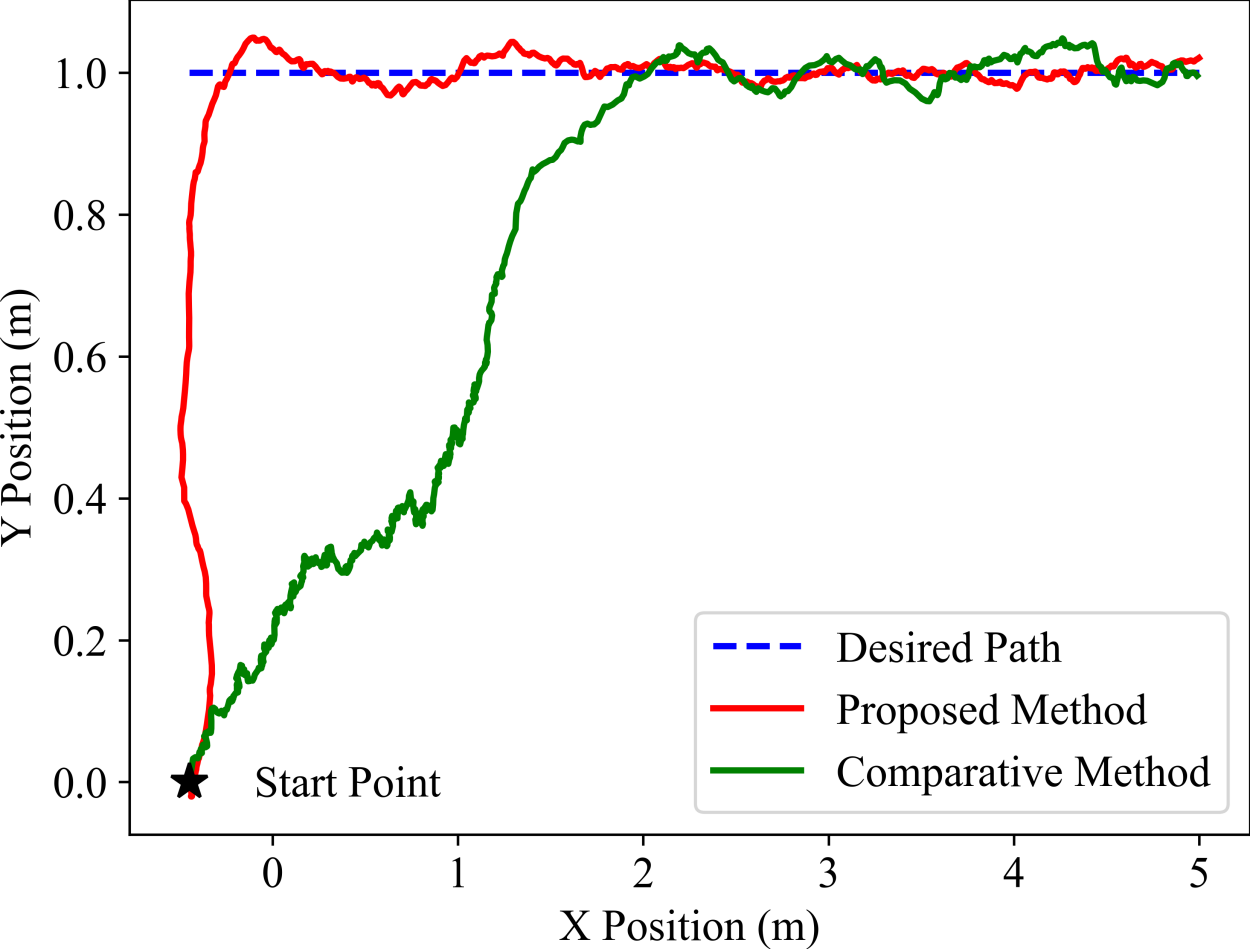
Simulation path following results of the desired line for the proposed hierarchical method and the end-to-end comparative method.

**Figure 4:**
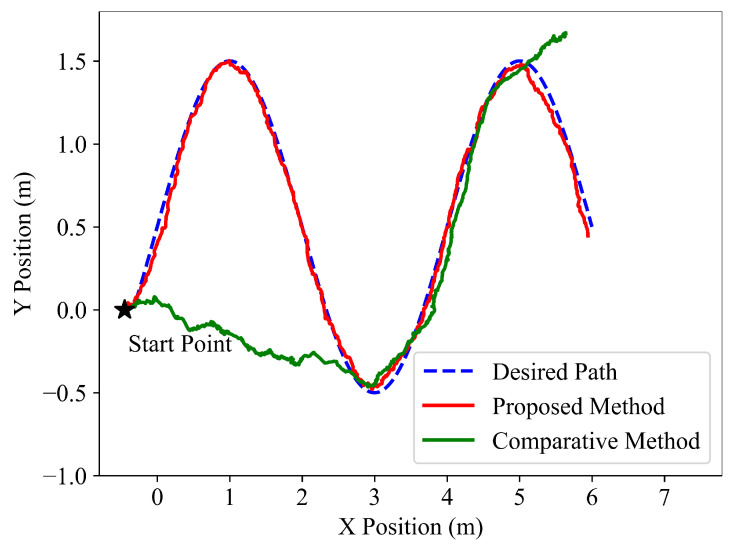
Simulation path following results of the desired sinusoidal curve for the proposed hierarchical method and the end-to-end comparative method.

**Figure 5:**
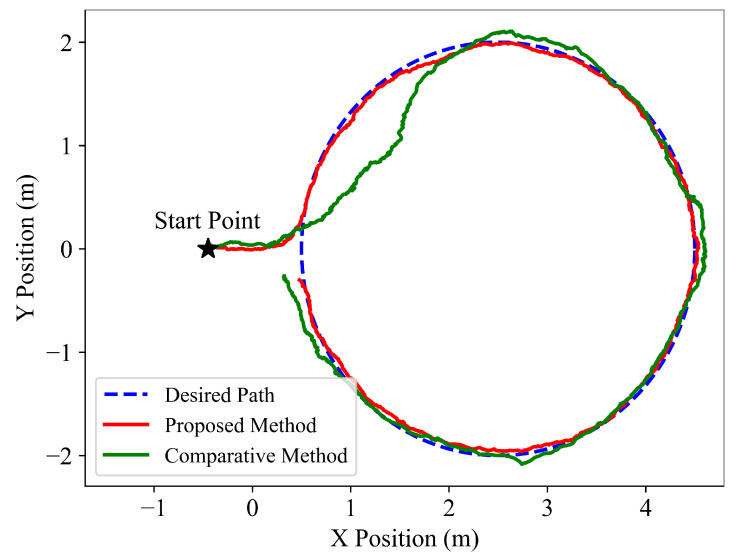
Simulation path following results of the desired circle for the proposed hierarchical method and the end-to-end comparative method.

To verify the following performance of the learned policy, the path following tests are performed on three different types of desired paths, and the following results are shown in Figure 3, Figure 4 and Figure 5. It is indicated that the snake robot converges to the desired path agilely and accurately, and then it keeps following the path with small tracking errors driven by the proposed method, implying the superior path following ability of the hierarchical trained policy; in comparison, under the control of the end-to-end comparative method, the snake robot requires longer convergence time and presents larger tracking errors.

### 3.2. Experiments

To validate the actual performance of the proposed method, two groups of hardware experiments are conducted on a self-built practical snake robot, and the results and analyses are provided in this part. Specifically, the snake robot is composed of visual localization module and motion modules, where the visual localization module contains a RealSense D435i camera for capturing images and a Dynamixel AX-12A actuator serving as the pan-tilt, and each 3D-printed body module has a mass of 0.416 g; it consists of a Hitec HS-5585MH actuator, a lithium battery pack with a voltage of 7.4 V, a wireless serial port and a pair of passive wheels.

**Figure 6:**
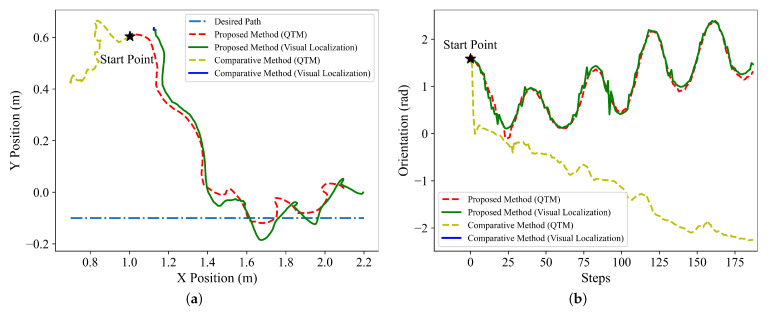
Results of visual localization for the trained policies with and without the visual stabilization item $p_{h}$. (**a**) Position results. (**b**) Orientation results.

Firstly, to examine whether visual localization stabilization term $p_{h}$ improves the localization accuracy by reducing head swings, we compare the accuracy of the visual localization for the trained policies with and without the stabilization item $p_{h}$, and the comparative results are depicted in Figure 6. To intuitively present the accuracy of the visual localization, the position and orientation results obtained by visual localization are compared with the results of the motion capture system Qualisys Track Manager (QTM). The desired path is y=−0.1, the start point of the snake robot is (1.0 m, 0.6 m), and the initial orientation is $\frac{\pi }{2}$.

From the results depicted in Figure 6, it is clear that the results of the visual localization are close to the actual results regardless of the position or orientation under the control of the proposed method. In contrast, under the control of the comparative method without the stabilization item $p_{h}$, the position and orientation of the snake robot (denoted by the solid blue lines shown in Figure 6) are only obtained at the beginning of the experiment, and subsequently, this information cannot be updated and the robot fails to follow the desired path, which is caused by the loss of visual target by the camera mounted on the head. At the beginning of the path following, the comparative method tends to generate a relatively large joint angle of the head to rapidly change the motion direction of the robot, thereby improving the convergence speed of the robot. However, the large head angle leads to a violent head swing, so the camera mounted on the head loses the visual target and thus, the pan-tilt cannot successfully compensate the joint angle of the head.

**Figure 7:**
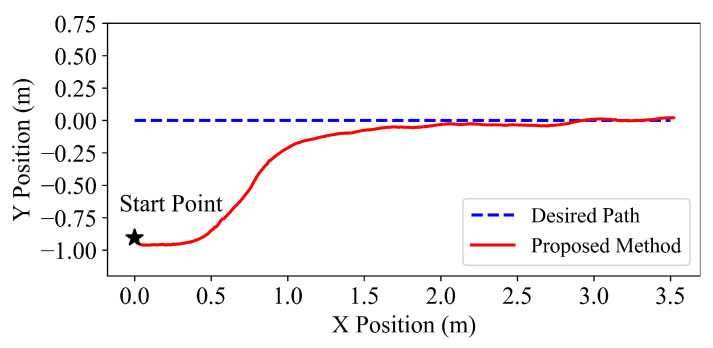
Path following results of the desired line for the proposed hierarchical method.

**Figure 8:**
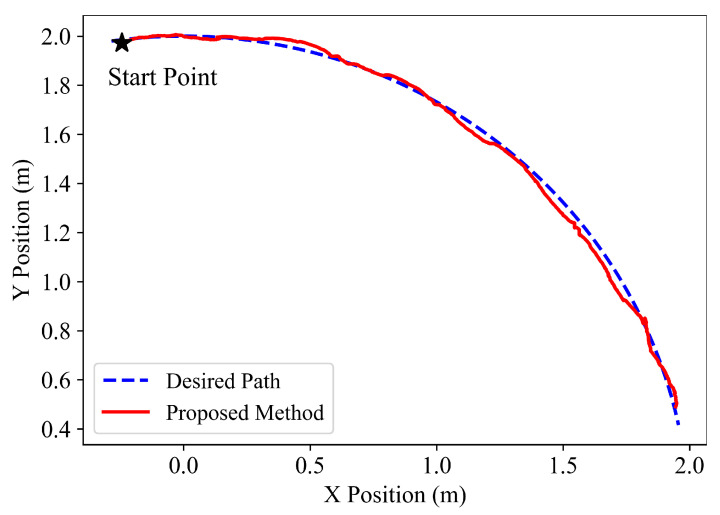
Path following results of the desired circle for the proposed hierarchical method.

Subsequently, different actual experiments for a straight line and a circle are carried out to evaluate the following accuracy of the proposed hierarchical path following method. For the straight-line path following, y=0.0 is taken as the desired path, and the start point of the snake robot is (0.0m,−0.9m). Then, circle $x^{2}+y^{2}=4$ is chosen as the desired path, and the start point is (−0.25m,2.0m). The following results are illustrated in Figure 7 and Figure 8, which denote that the learned hierarchical path following policy can be directly transferred to the practical system and successfully drive the snake robot to converge to and then follow the desired paths.

## 4. Discussion

We can observe that the experimental results shown in Figure 8 exhibit the following performance of a snake robot for a quarter circle rather than an entire circle. This is because the number of the visual marker and the maximum rotation angle of the pan-tilt in our experiment are both limited; that is, there is only one fixed visual marker, and the rotation angle of the pan-tilt is limited to (−140°, 140°). When the snake robot tracks the remaining three-quarters of the circle, the direction of the robot head will be opposite to the direction of the visual marker so that the angle that the pan-tilt needs to compensate for is larger than the maximum rotation angle, and the visual localization cannot be successfully completed.

Additionally, it is noteworthy that the accuracy of the path following in simulations and experiments mainly depends on the accuracy of localization. However, in this paper, an external visual marker is employed to assist positioning, which introduces additional systematic errors. In addition, the lack of diversity of the input data sources, i.e., only a monocular camera, limits the robustness and stability of the localization.

Future work: In future, we will focus on more intelligent methods to solve the problems mentioned above. Firstly, we will adopt multiple visual markers to assist the visual localization. When the direction of the head changes, a snake robot will autonomously select a visual marker in the corresponding direction to update its position so as to achieve the all-around visual positioning and track the path in any direction. In addition, we will focus on multi-sensor fusion technologies, where the sensors include GPS, IMU, camera, radar, etc., to complete autonomous perception without any external assistance, which will further improve the intelligence of perception and expand the application scenarios of snake robots. Finally, in order to further improve the robustness of the proposed method, some extrinsic perturbations will be imposed on a snake robot during training, including external force disturbance, sensory information noise and changes in physical parameters, thereby promoting the adaptability of a snake robot to system errors and changes in different application scenarios.

## 5. Conclusions

In this paper, a hierarchical RL-based control method is proposed to achieve satisfactory path following performance for snake robots with an onboard camera. Specifically, firstly, the hierarchical path following method, consisting of the RL policy training layer and the gait executive layer, optimizes the learning efficiency, exhibits reliable path following ability, and guarantees the transferability of the learned policy to the practical system by sufficiently combining the advantage of the RL policy network and the gait equation. Subsequently, the position of a snake robot can be updated in real time via visual localization due to the introduction of the visual localization stabilization item in the reward function. A series of simulation and hardware experimental results validate that the proposed method is capable of achieving a precise and fast convergence with respect to the path following tasks for a snake robot with autonomous visual perception.

## Data Availability

Not applicable.
